# Prediction of HIV sensitivity to monoclonal antibodies using aminoacid sequences and deep learning

**DOI:** 10.1093/bioinformatics/btac530

**Published:** 2022-07-25

**Authors:** Vlad-Rareş Dănăilă, Cătălin Buiu

**Affiliations:** Department of Automatic Control and Systems Engineering, Politehnica University of Bucharest, Bucharest 060042, Romania; Department of Automatic Control and Systems Engineering, Politehnica University of Bucharest, Bucharest 060042, Romania

## Abstract

**Motivation:**

Knowing the sensitivity of a viral strain versus a monoclonal antibody is of interest for HIV vaccine development and therapy. The HIV strains vary in their resistance to antibodies, and the accurate prediction of virus-antibody sensitivity can be used to find potent antibody combinations that broadly neutralize multiple and diverse HIV strains. Sensitivity prediction can be combined with other methods such as generative algorithms to design novel antibodies *in silico* or with feature selection to uncover the sites of interest in the sequence. However, these tools are limited in the absence of *in silico* accurate prediction methods.

**Results:**

Our method leverages the CATNAP dataset, probably the most comprehensive collection of HIV-antibodies assays, and predicts the antibody-virus sensitivity in the form of binary classification. The methods proposed by others focus primarily on analyzing the virus sequences. However, our article demonstrates the advantages gained by modeling the antibody-virus sensitivity as a function of both virus and antibody sequences. The input is formed by the virus envelope and the antibody variable region aminoacid sequences. No structural features are required, which makes our system very practical, given that sequence data is more common than structures. We compare with two other state-of-the-art methods that leverage the same dataset and use sequence data only. Our approach, based on neuronal networks and transfer learning, measures increased predictive performance as measured on a set of 31 specific broadly neutralizing antibodies.

**Availability and implementation:**

https://github.com/vlad-danaila/deep_hiv_ab_pred/tree/fc-att-fix

## 1 Introduction

HIV is characterized by a high mutation rate, enabling the virus to adapt rapidly and to circulate under diverse strains. Some of the strains are neutralized by the antibodies, but some resistant ones remain and continue the infection. Due to HIV diversity, combinations of broadly neutralizing antibodies are more likely to be efficient than a single antibody in combating the virus (Williamson *et al.*, 2021a). In addition to the potency of neutralization, the breadth of neutralization, or how many strains can be neutralized by a particular antibody is essential, and some works focus on this aspect (Cheng *et al.*, 2018; Conti and Karplus, 2019; Sevy *et al.*, 2018; Williamson *et al.*, 2021a; Yu *et al.*, 2019). A model that can accurately determine the neutralization potency for a given antibody-virus pair can be useful for the analysis of neutralization coverage and for finding ideal antibody combinations.

The neutralization potency was predicted by machine learning techniques in Hepler *et al.* (2014), Buiu *et al.* (2016), Hake and Pfeifer (2017), Rawi *et al.* (2019), Conti and Karplus (2019), Yu *et al.* (2019), Magaret *et al.* (2019) and Williamson *et al.* (2021a). SLAPNAP (Williamson *et al.*, 2021a) predicts the neutralization of specific antibodies with more predictors: elastic net (Zou and Hastie, 2005), random forests (RF) (Breiman, 2001), gradient boosted machines (GBM) (Friedman, 2001) and extreme gradient boosting (XGBoost) (Chen and Guestrin, 2016). The user can choose a predictor or combine more of them using an ensemble named Super Learner (van der Laan *et al.*, 2007). In addition, SLAPNAP calculates the importance of features and predicts the neutralization of combinations of antibodies using either an additive or Bliss-Hill model (Wagh *et al.*, 2016). GBM (Friedman, 2001) was used in Rawi *et al.* (2019) to predict the sensitivity of viruses to 33 antibodies from the CATNAP database (Yoon *et al.*, 2015). The input consisted of one-hot encoded virus aminoacid sequences (Rawi *et al.*, 2019). The GBM outperformed other algorithms, such as logistic regression, RF and the support vector machine (SVM) from Hake and Pfeifer (2017). In Hake and Pfeifer (2017), an SVM with string kernels (Meinicke *et al.*, 2004; Rätsch *et al.*, 2005) was compared against RF (Liaw *et al.*, 2002), a neural network, least absolute shrinkage and selection operator (LASSO) (Friedman *et al.*, 2010), and a linear SVM (Karatzoglou *et al.*, 2004). The virus neutralization was determined for eleven selected antibodies and the measurements uncovered an increase of virus resistance in time (Hake and Pfeifer, 2017). In Conti and Karplus (2019), neural networks (NNs) with one or two layers, k-nearest neighbors (Altman, 1992), RF (Ho, 1995; Svetnik *et al.*, 2003) and SVM (Cortes and Vapnik, 1995) receives an input of atomistic descriptors and predicts the potency of antibodies that target the highly conserved CD4 region. The glycans that cover the virus envelope play an essential role in the interaction with antibodies, and Yu *et al.* (2018) used a system composed of Metropolis-Hastings algorithm (Andrieu *et al.*, 2003; Hastings, 1970) and support vector regression (SVR) (Cortes and Vapnik, 1995; Drucker *et al.*, 1997) to assess the importance of specific glycans and protein sites for antibody binding. This system is used in Yu *et al.* (2019) as well for feature selection prior to regression of the neutralization sensitivity. HIV neutralization and feature importance was studied for a singular broadly neutralizing antibody VRC01 in Magaret *et al.* (2019) using LASSO (Tibshirani, 1996), RF (Liaw *et al.*, 2002), Naïve Bayes (John and Langley, 1995), XGBoost (Chen and Guestrin, 2016), generalized linear models and an ensemble named Super Learner (van der Laan *et al.*, 2007). Buiu *et al.* (2016) regressed the neutralization measures for a panel of selected antibody-virus pairs using NN. B-cell receptor sequence repertoires were analyzed using phylogenetic trees for uncovering potentially effective antibodies and determining favorable mutations in Ralph and Matsen (2020).

The detection of epitopes, which are sites on the antigen bound by the antibody, is an important study topic for vaccine design and is sometimes analyzed together with virus neutralization potency. Some of the works focused on epitope detection are Gnanakaran *et al.* (2010), Wang *et al.* (2011), Ren *et al.* (2014), Evans *et al.* (2014), Hepler *et al.* (2014), Nogal *et al.* (2017), Cheng *et al.* (2018), Yu *et al.* (2018), Bricault *et al.* (2019), Magaret *et al.* (2019), Rawi *et al.* (2019), Kaku *et al.* (2020), Ralph and Matsen (2020) and Williamson *et al.* (2021a). In the current material, we are not investigating epitope detection.

## 2 Approach

Most authors (Buiu *et al.*, 2016; Hake and Pfeifer, 2017; Hepler *et al.*, 2014; Magaret *et al.*, 2019; Rawi *et al.*, 2019; Williamson *et al.*, 2021a; Yu *et al.*, 2019) create multiple classifiers/regressors, and each of those models is trained with a subset of viruses as input and the outcomes specific to a certain antibody as ground truths. For example, if the dataset contained assays specific to ten antibodies, ten separate models are trained, one for each antibody. If the neutralization potency of a combination of antibodies against a virus needs to be estimated, that is achieved by combining the estimations from the models trained on each antibody. CATNAP also provides assays for certain combinations of antibodies, which can be used for validation. SLAPNAP (Williamson *et al.*, 2021a) took this approach to predict the potency of antibody cocktails by leveraging an additive and a Bliss-Hill model (Wagh *et al.*, 2016).

The sequences of the virus envelopes are used as input without taking into account the antibodies sequences. This has the advantages of lowering the feature space dimensionality and simpler modeling. We take a different approach and use both antibody and antigen sequences at once as input to our model. Our rationale is that more generic interactions can be modeled this way. Moreover, we can leverage substantially more data, ∼32 000 combinations of antigen-antibody sequence pairs. In contrast, when grouping viruses by antibodies, the data amount is reduced to hundreds of samples per antibody at best. Therefore, if an antibody has too little data available, it becomes impossible to analyze with the previous approaches; however, our setup does not have this drawback. Using both antigen and antibody sequences and NNs, we can take advantage of transfer learning to pretrain on the majority of the antibody-antigen pairs and fine-tune the model on specific antibodies of interest. This is an essential advantage provided by NNs that would not be possible with the decision-tree or SVM-based algorithms mentioned in Section 1.

**Fig. 1.**
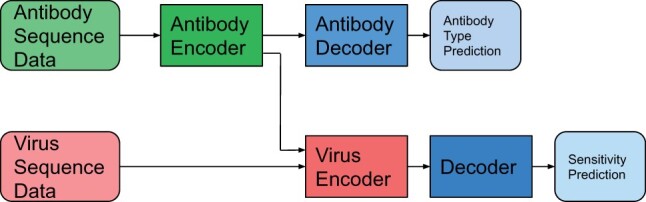
The system architecture. The antibody decoder used for antibody type prediction is applied only for multitasking

As shown in Figure 1, the architecture of our system consists of:

- a module that encodes antibody sequences
- a module that analyzes the virus sequence and the encoded antibody
- a decoder

Each module can take multiple forms, as described in Section 3, we experimented with GRU (Cho *et al.*, 2014), fully connected layers, attention, transformers (Vaswani *et al.*, 2017), more input encoding strategies and multitask learning.

Since Rawi *et al.* (2019) and Williamson *et al.* (2021a) report state-of-the-art results in virus neutralization binary classification, we compare with those works and display significant improvements in terms of recorded accuracy, Matthews correlation coefficient (MCC) and receiver operating characteristic area under the curve (AUC).

## 3 Materials and methods

Due to the costly nature of training NNs, we did not perform an exhaustive search across the combinations of models, input processing and hyperparameters. Despite this, in our search, we came across several configurations that measured promising results. To keep the article concise, we document only the most representative models.

### 3.1 Models

In this subsection, we elaborate on the models’ structures and architectures. In all variations, the decoder consists of a fully connected layer with dropout and sigmoid activation; however, the encoders and input will vary. For avoiding overfitting, the GRU, transformers and all fully connected networks are only one layer deep.

1. ICERI—GRU encoders for both antibody and virus: in the current article, we are building on top of our previous work (Dănăilă and Buiu, 2021), where we processed the antibody light chain, heavy chain and the virus envelope sequences by three GRUs (Cho *et al.*, 2014) to classify viruses as resistant or sensitive to a particular antibody. The hidden states resulting from running the light chain and heavy chain-specific GRUs are concatenated and form the initial hidden state of the virus GRU (Dănăilă and Buiu, 2021).
2. FC-ATT-GRU—Fully connected and attention for antibody and GRU for virus: each of the antibody light and heavy chains is processed by a separate module consisting of a fully connected layer, dropout and attention as in Algorithm 1. The light and heavy chains encodings are concatenated and form the initial hidden state of the GRU. The GRU receives as input the virus envelope sequence.
3. 6CDR-FC-GRU—Fully connected for complementary determining regions (CDR) and GRU for virus: in this case, we do not consider the complete sequence, only the six CDRs. Each CDR is encoded by a separate fully connected layer and dropout. These encodings are concatenated and form the initial hidden state of the virus processing GRU network. We do not use attention since the CDRs are implicitly the most important regions.
4. TRANSF—Transformers (Vaswani *et al.*, 2017): the antibody sequence is input to the encoder part of the transformer and the virus sequence to the decoder. The resulting feature vector is processed by a fully connected layer to predict the binary outcome.
5. MULTITASK: it is the same model as FC-ATT-GRU, but trained with multitasking.

### 3.2 Data preprocessing

The aminoacid sequences are strings containing 22 letters, 20 denote the DNA encoded aminoacids, ‘-’ for gaps and ‘X’ for unknown elements. For encoding each aminoacid element, we used the following methods: learned embeddings, one-hot-encodings and a vector of size seven that summarizes the properties of the aminoacid (Meiler *et al.*, 2001).

The potential N-linked glycosylation sites (PNGS) are of significant importance for modeling the antibody-antigen interactions (Yu *et al.*, 2019). In the current work, as well in Dănăilă and Buiu (2021), we represent PNGS as a binary mask that we concatenate to the virus sequence features.

Similarly to Dănăilă and Buiu (2021), every time we used GRU networks, the input consisted of encoded k-mers, which were overlapping substrings of length k from the aminoacid sequence. Therefore, each step of the sequence fed to the GRU consisted of a k-mer. Other works that used k-mers are Ren *et al.* (2014), Wang *et al.* (2011), Choi *et al.* (2015), Gnanakaran *et al.* (2010) as cited in Dănăilă and Buiu (2021). The length and stride of the k-mers were established as in Section 3.4. If the data were input to fully connected layers or transformers, k-mers were not used anymore. PNGS binary masks were transformed in k-mers as well and concatenated with the aminoacid sequence k-mers (Dănăilă and Buiu, 2021).

For models using CDRs, each of the six CDRs was modeled by a numeric array encompassing the aminoacids of the CDR and a continuous value denoting the position of the CDR inside the sequence as in Algorithm 2. We used Paratome (Kunik *et al.*, 2012) and AbRSA (Li *et al.*, 2019) to find the antibodies CDRs sites.

For transformers, we constrained the antibody input sequence to the sites between 17 to 77 and 84 to 133 for the light chain and from 13 to 79 and 83 to 135 for the heavy chain to reduce the data dimensionality. The intervals were established based on the minimum and maximum positions of the CDRs aminoacids as found through Paratome (Kunik *et al.*, 2012) and AbRSA (Li *et al.*, 2019).

The binary outcomes (ground truth) were determined by comparing the IC50 (half maximal inhibitory concentration) with a threshold, which in our experiments was 50, as in Rawi *et al.* (2019) and Williamson *et al.* (2021a). However, in some CATNAP assays, the IC50 was expressed as censored values, which means that the precise quantity is unknown, only that it is less or more than a certain threshold; the most frequent censored quantity is ‘>50’. Also, for some antibody-virus combinations, there are recorded multiple IC50 values, some can be exact, and others censored. For such cases, we estimated the mean IC50 using a popular method for censored regression, the Tobit model (Amemiya, 1984; Olsen, 1978; Tobin, 1958). Our implementation of the Tobit model is based on PyTorch and optimized through gradient descent.

Algorithm 1 Processing of antibody sequence by fully connected layers

**function** encode_antibody(ab_sequence)

 $att_{fc}=\mathrm{attention}\_\mathrm{fully}\_\mathrm{connected}\_\mathrm{layer}(ab\_\mathrm{sequence})$

 $att_{fc\_\mathrm{drop}}=\mathrm{attention}\_\mathrm{dropout}(att_{fc})$

 $\mathrm{attention}=\mathrm{sigmoid}(att_{fc\_\mathrm{drop}})$

 $ab_{fc}=ab\_\mathrm{fully}\_\mathrm{connected}\_\mathrm{layer}(ab\_\mathrm{sequence})$

 $ab_{fc\_\mathrm{drop}}=ab\_\mathrm{dropout}(ab_{fc})$

 $\mathrm{return}\;\;\mathrm{attention}\cdot ab_{fc\_\mathrm{drop}}$

**end function**

Algorithm 2 Processing of the 6 CDRs sequences of a given antibody.

*seq* = list containing the aminoacid strings of the 6 CDRs

max_sizes = list with the maximum size for each of the 6 CDRs

*pos* = list with the positions of the centers of the 6 CDRs

pos_mean = list with the average position of the centers of the 6 CDRs

pos_std = list with the positions std of the centers of the 6 CDRs

**function** pad_sequence(*sequence*, max_size)

 *sequence* is padded to the left and to the right (centered), with a particular character denoting unknown aminoacids so that we obtain a sequence of length max_size.

**end function**

*result* = empty array of size 6

**for**  i=0,…5  **do**

 *seq_padded_* = *pad_sequence*(*seq*[*i*], *max_sizes*[*i*])

 $pos_{\mathrm{normalized}}=(\mathrm{pos}[i]-\mathrm{pos}\_\mathrm{mean}[i])/\mathrm{pos}\_\mathrm{std}[i]$

 $\mathrm{result}[i]=\mathrm{concatenate}(seq_{\mathrm{padded}},pos_{\mathrm{normalized}})$

**end for**

**return** *result*

### 3.3 Optimization

For optimization, the PyTorch RMSprop was used in all cases, except for training the transformers, where we used the Noam optimizer (Vaswani *et al.*, 2017).

### 3.4 Hyperparameter optimization

The hyperparameters, such as k-mer length and stride (see Section 3.2), batch size, learning rate, gradient clip, dropout rates, and parameters defining the network structure were found automatically, through hyperparameter optimization, using the Optuna implementation of TPE (Tree-structured Parzen Estimator) (Bergstra *et al.*, 2011). In all cases, the TPE was univariate, except for transformers when it was multivariate, see the TPE documentation. For efficiency, we employed a pruner that interrupted the training for unpromising experiments based on intermediary results or for those taking too much time.

### 3.5 Multitask learning

In the multitask setting, the entire network is trained to predict the virus-antibody sensitivity, and the antibody encoder is attached a fully connected layer (antibody decoder) to classify the type of antibody as in Figure 1. Some antibodies can belong to multiple classes. The two tasks are trained simultaneously, having as loss a weighted sum of two binary cross-entropies.

## 4 Results

We are comparing with bNAb-ReP (Rawi *et al.*, 2019) and SLAPNAP (Williamson *et al.*, 2021a). The two were also compared in Williamson *et al.* (2021a), and bNAb-ReP recorded a median AUC of 0.84 and SLAPNAP of 0.81; however, the two were not evaluated in the same way. In our work, we also look at MCC, which is a more discriminative metric than AUC.

In bNAb-ReP, the hyperparameters of the GBM were found through grid search (Rawi *et al.*, 2019) and the model was evaluated by repeating for ten times a cross-validation having 10 folds (Rawi *et al.*, 2019).

In SLAPNAP, the Super Learner (van der Laan *et al.*, 2007) model is trained and evaluated on one round of five-fold cross-validation (Williamson *et al.*, 2021a). However, the Super Learner algorithm performs automatic hyperparameter optimization based on cross-validation as part of its’ training process (Williamson *et al.*, 2021a). Therefore, in SLAPNAP, nested cross-validation is happening, the inner cross-validation is used for hyperparameter optimization, and the outer cross-validation is used for evaluation (Williamson *et al.*, 2021a). The test data from the outer cross-validation folds is not found in any of the folds used for inner cross-validation; therefore, the evaluation data is completely uncoupled from the rest of the dataset.

For comparing with the other works, we follow similar evaluation procedures as in the compared papers, repeated cross-validation for bNAb-ReP and nested cross-validation for SLAPNAP. In addition, we pretrain on the CATNAP data. We also optimize the hyperparameters in two parts. Part one is related to pretraining on CATNAP and finding the network structure. The second part is specific to each antibody and aims to find the learning parameters such as batch size, learning rate, gradient clip and dropout rates. Part two was performed in 1000 iterations per antibody and used cross-validation. Due to the larger size of the dataset, part one optimization was performed in over 400 iterations using a training/validation split of CATNAP. In both cases, the hyperparameter optimization is performed by the TPE algorithm (Bergstra *et al.*, 2011), as described in Section 3.4, by maximizing the MCC. The antibody-specific training occurs only for the virus encoder and decoder, while all parts for handling antibody data remain frozen. In all training procedures, we employed early stopping by selecting the model with the highest MCC from all epochs. Algorithm 3 displays the complete procedures for comparing with both works, which include pretraining and antibody specific fine-tuning.

Algorithm 3 Comparison with bNAb-ReP in function evaluate_by_repeated_cross_validation and with SLAPNAP in function evaluate_by_nested_cross_validation.

**function** hyperparam_opt(*data*, *model*)

 hyperparameter tuning of *model*

 *data* is split into training and validation sets

 **return** *hyperparameters*

**end function**

**function** cross_val_hyperparam(*data*, *model*)

 cross validated hyperparameter tuning of *model* using *data*

 **return** *hyperparameters*

**end function**

**function** pretrain(*data*, *hyperparam*, *model*)

 train *model* on *data* using *hyperparam*

 **return** trained *model*

**end function**

**function** cross_validate(*data*, *hyperparam*, *model*)

 cross validate *model* on *data* using *hyperparam*

 **return** *metrics*

**end function**

**function** train_test(*data_train_*, *data_test_*, *hyperparam*, *model*)

 train *model* on *data_train_* using *hyperparam*

 evaluate *model* on *data_test_* using *hyperparam*

 **return** *metrics*

**end function**

*catnap* = data from CATNAP

*model* = a non-trained model

*param_pretr_* = hyperparam_opt(*catnap*, *model*)

**function** evaluate_by_repeated_cross_validation

 **for** *antibody* in evaluated_antibodies  **do**

  *data_cv_* = select all in *catnap* containing *antibody*

  *data_pretr_* = select all in *catnap* not containing *antibody*

  *model_pretr_* = pretrain(*data_pretr_*, *param_pretr_*, *model*)

  *param_cv_* = cross_val_hyperparam(*data_cv_*, *model_pretr_*)

  *metrics* = empty array

  **for**  i=0…9  **do**

   *metrics_cv_* = cross_validate(*data_cv_*, *param_cv_*, *model_pretr_*)

   append *metrics_cv_* to *metrics*

  **end for**

  **record** mean of *metrics*

 **end for**

**end function**

**function** evaluate_by_nested_cross_validation

 outer_cross_valid = list of train/test dataset partitions

 metrics_matrix = empty matrix of size 5 (folds) by 32 (antibodies)

 **for** *data_train_*, *data_test_* in outer_cross_valid  **do**

  **for** *antibody* in evaluated_antibodies  **do**

   *data_pretr_* = select all in *catnap* not containing *antibody*

   *model_pretr_* = pretrain(*data_pretr_*, *param_pretr_*, *model*)

   *param_cv_* = cross_val_hyperparam(*data_train_*, *model_pretr_*)

   *m* = train_test(*data_train_*, *data_test_*, *param_cv_*, *model_pretr_*)

   insert *m* into metrics_matrix

  **end for**

 **end for**

 **record** means per antibodies from metrics_matrix

**end function**

Comparing with the other works is a costly operation because it implies fine-tuning for each antibody. Therefore, we resorted to a simplified method to select the best model architecture. We first found the ideal hyperparameters on CATNAP. Then, for each antibody in bNAb-ReP, we trained on the rest of CATNAP (excluding the records having that antibody) and evaluated using the data containing that antibody. This is similar to the procedure used for comparing with the other works but without fine-tuning per antibody. Table 1 displays the results for the model selection. All models had similar results, and the best MCC is recorded for FC-ATT-GRU and 6CDR-FC-GRU. Both networks had aminoacid properties as input. Between the two, we selected FC-ATT-GRU for comparison with bNAb-ReP and SLAPNAP because it is a more practical model; determining the CDR complicates the input processing while providing only a minor performance advantage. Also, an input formed out of the aminoacid properties shows better performance while lowering the dimensions of the tensors and speeding up the computation.

**Table 1.** Metrics averaged across the bNAb-ReP antibodies for the pretrained models (without antibody specific fine-tuning)

Model	Input	MCC	AUC	Accuracy
ICERI	Learned embeddings	0.5534	0.8207	0.7945
ICERI	One-hot	0.5573	0.8314	0.7946
ICERI	Aminoacid properties	0.5587	0.8301	0.8061
ICERI	One-hot & aminoacid properties	0.5518	0.8237	0.8054
FC-ATT-GRU	One-hot	0.5569	0.8222	0.7970
FC-ATT-GRU	Aminoacid properties	0.5770	0.8378	0.8097
FC-ATT-GRU	One-hot & aminoacid properties	0.5651	0.8321	0.7972
6CDR-FC-GRU	Aminoacid properties	0.5777	0.8391	0.7956
TRANSF	Aminoacid properties	0.5640	0.8277	0.8033
MULTITASK	Aminoacid properties	0.5682	0.8402	0.7971

Table 2 shows the results for the finetuned FC-ATT-GRU model versus bNAb-ReP, and Table 3 compares FC-ATT-GRU with SLAPNAP.

**Table 2.** Comparison with bNAb-ReP on 100 rounds of cross-validation (10 folds cross-validation repeated 10 times)

	bNAb-ReP			FC-ATT-GRU		
Antibody	MCC	AUC	Accuracy	MCC	AUC	Accuracy
gp120 CD4BS						

3BNC117	0.69 (0.13)	0.88 (0.07)	0.90 (0.04)	**0.77 (0.10)**	**0.91 (0.06)**	**0.92 (0.04)**
b12	**0.56 (0.11)**	**0.82 (0.05)**	0.79 (0.07)	0.55 (0.08)	0.81 (0.05)	**0.80 (0.04)**
HJ16	0.42 (0.15)	0.67 (0.11)	0.66 (0.14)	**0.47 (0.13)**	**0.70 (0.11)**	**0.76 (0.09)**
NIH45-46	0.59 (0.15)	0.80 (0.14)	0.89 (0.05)	**0.87 (0.12)**	**0.94 (0.08)**	**0.96 (0.04)**
VRC-CH31	0.60 (0.16)	0.78 (0.15)	0.87 (0.06)	**0.79 (0.14)**	**0.89 (0.09)**	**0.93 (0.05)**
VRC-PG04	0.57 (0.15)	0.78 (0.10)	0.87 (0.06)	**0.84 (0.10)**	**0.95 (0.04)**	**0.94 (0.04)**
VRC01	0.70 (0.12)	0.89 (0.07)	0.92 (0.03)	**0.81 (0.08)**	**0.93 (0.05)**	**0.94 (0.02)**
VRC03	0.61 (0.14)	0.83 (0.08)	0.81 (0.07)	**0.75 (0.11)**	**0.88 (0.06)**	**0.87 (0.05)**
VRC07	0.66 (0.16)	0.78 (0.16)	0.93 (0.04)	**0.83 (0.16)**	**0.93 (0.12)**	**0.95 (0.10)**
Average	0.60 (0.14)	0.80 (0.10)	0.84 (0.06)	**0.74 (0.11)**	**0.88 (0.07)**	**0.90 (0.05)**

gp120 other than CD4BS						

10-1074	0.86 (0.08)	0.95 (0.04)	0.94 (0.04)	**0.93 (0.05)**	**0.98 (0.02)**	**0.96 (0.03)**
2G12	**0.75 (0.10)**	**0.93 (0.05)**	**0.91 (0.04)**	0.63 (0.10)	0.88 (0.06)	0.87 (0.04)
CH01	0.56 (0.16)	0.77 (0.10)	0.77 (0.08)	**0.76 (0.10)**	**0.88 (0.06)**	**0.87 (0.05)**
DH270.1	0.82 (0.12)	0.92 (0.07)	0.90 (0.07)	**0.87 (0.09)**	**0.95 (0.04)**	**0.93 (0.05)**
DH270.5	0.83 (0.11)	0.93 (0.05)	0.91 (0.06)	**0.93 (0.08)**	**0.98 (0.03)**	**0.96 (0.04)**
DH270.6	0.85 (0.12)	0.93 (0.07)	0.93 (0.06)	**0.91 (0.09)**	**0.97 (0.03)**	**0.95 (0.05)**
PG16	0.57 (0.13)	0.79 (0.08)	0.84 (0.05)	**0.67 (0.12)**	**0.85 (0.06)**	**0.86 (0.06)**
PG9	0.61 (0.12)	0.85 (0.07)	0.86 (0.04)	**0.78 (0.08)**	**0.92 (0.04)**	**0.91 (0.03)**
PGDM1400	0.66 (0.12)	0.83 (0.10)	0.89 (0.05)	**0.82 (0.09)**	**0.95 (0.03)**	**0.93 (0.04)**
PGT121	0.75 (0.10)	0.92 (0.05)	0.88 (0.05)	**0.84 (0.08)**	**0.95 (0.03)**	**0.92 (0.04)**
PGT128	0.72 (0.08)	0.89 (0.05)	0.86 (0.04)	**0.77 (0.08)**	**0.91 (0.04)**	**0.88 (0.04)**
PGT135	0.54 (0.13)	0.77 (0.09)	0.74 (0.10)	**0.63 (0.11)**	**0.82 (0.09)**	**0.81 (0.06)**
PGT145	0.67 (0.11)	0.86 (0.06)	0.86 (0.05)	**0.70 (0.11)**	**0.88 (0.06)**	**0.86 (0.05)**
VRC26.08	0.70 (0.10)	0.89 (0.05)	0.85 (0.05)	**0.90 (0.06)**	**0.97 (0.02)**	**0.95 (0.03)**
VRC26.25	0.71 (0.13)	0.89 (0.06)	0.87 (0.06)	**0.88 (0.08)**	**0.96 (0.03)**	**0.94 (0.04)**
VRC38.01	**0.70 (0.14)**	**0.87 (0.08)**	**0.87 (0.07)**	0.66 (0.14)	0.83 (0.10)	0.86 (0.07)
Average	0.70 (0.12)	0.87 (0.07)	0.86 (0.06)	**0.79 (0.09)**	**0.92 (0.05)**	**0.90 (0.04)**

gp41 MPER, gp41-gp120 interface, and fusion peptide						

2F5	**0.89 (0.05)**	**0.97 (0.02)**	**0.95 (0.03)**	0.83 (0.06)	0.94 (0.03)	0.91 (0.03)
35O22	0.38 (0.13)	0.63 (0.11)	0.66 (0.10)	**0.48 (0.14)**	**0.70 (0.10)**	**0.73 (0.07)**
4E10	**0.63 (0.13)**	**0.82 (0.12)**	**0.94 (0.03)**	0.61 (0.14)	0.78 (0.12)	0.93 (0.03)
8ANC195	**0.77 (0.11)**	**0.90 (0.07)**	**0.89 (0.05)**	0.58 (0.12)	0.78 (0.09)	0.79 (0.07)
PGT151	0.58 (0.13)	0.78 (0.09)	0.83 (0.06)	**0.71 (0.13)**	**0.87 (0.08)**	**0.87 (0.08)**
VRC34.01	**0.61 (0.15)**	**0.78 (0.10)**	**0.79 (0.08)**	0.51 (0.12)	0.71 (0.11)	0.73 (0.08)
Average	**0.64 (0.12)**	**0.81 (0.08)**	**0.84 (0.06)**	0.62 (0.12)	0.79 (0.09)	0.83 (0.06)

Global average	0.66 (0.12)	0.84 (0.08)	0.85 (0.06)	**0.75 (0.10)**	**0.88 (0.06)**	**0.89 (0.05)**

*Note*: The numbers between the parentheses are the standard deviations (calculated with N-1 degrees of freedom) of the metrics recorded on the 100 rounds of cross-validation. The bNAb-ReP metrics are taken from the Supplementary Table S1 from Rawi *et al.* (2019), except for the standard deviations, which we recomputed by running the bNAb-ReP software to ensure the same calculation method as in our work. The boldface values highlight the best metrics between bNAb-ReP and our model.

**Table 3.** Comparison with SLAPNAP on nested cross-validation

	SLAPNAP			FC-ATT-GRU		
Antibody	MCC	AUC	Accuracy	MCC	AUC	Accuracy
gp120 CD4BS						

3BNC117	0.06 (0.14)	0.80 (0.05)	0.93 (0.00)	**0.76 (0.04)**	**0.92 (0.03)**	**0.93 (0.01)**
b12	0.43 (0.06)	0.78 (0.05)	0.71 (0.03)	**0.51 (0.05)**	**0.79 (0.03)**	**0.79 (0.02)**
HJ16	**0.41 (0.09)**	**0.77 (0.03)**	**0.72 (0.04)**	0.24 (0.11)	0.63 (0.11)	0.58 (0.13)
NIH45-46	0.21 (0.22)	0.82 (0.13)	0.88 (0.02)	**0.86 (0.07)**	**0.96 (0.04)**	**0.96 (0.02)**
VRC-CH31	0.12 (0.17)	0.74 (0.05)	0.85 (0.01)	**0.79 (0.10)**	**0.93 (0.05)**	**0.94 (0.02)**
VRC-PG04	0.05 (0.15)	0.80 (0.05)	0.84 (0.01)	**0.79 (0.05)**	**0.94 (0.03)**	**0.92 (0.03)**
VRC01	0.30 (0.30)	0.77 (0.10)	**0.96 (0.01)**	**0.81 (0.06)**	**0.92 (0.05)**	0.95 (0.02)
VRC03	0.41 (0.12)	0.83 (0.05)	0.75 (0.04)	**0.70 (0.05)**	**0.89 (0.02)**	**0.84 (0.02)**
VRC07	0.00 (0.01)	0.78 (0.17)	**0.95 (0.01)**	**0.71 (0.12)**	**0.91 (0.07)**	0.94 (0.02)
Average	0.22 (0.14)	0.79 (0.08)	0.84 (0.02)	**0.69 (0.07)**	**0.88 (0.05)**	**0.87 (0.03)**

gp120 other than CD4BS						

10-1074	0.77 (0.08)	0.92 (0.02)	0.91 (0.03)	**0.90 (0.05)**	**0.98 (0.01)**	**0.95 (0.02)**
10-996	0.74 (0.10)	0.93 (0.07)	0.87 (0.05)	**0.90 (0.13)**	**0.98 (0.01)**	**0.95 (0.07)**
2G12	0.47 (0.03)	0.82 (0.01)	0.75 (0.02)	**0.61 (0.10)**	**0.86 (0.03)**	**0.87 (0.04)**
CH01	0.47 (0.08)	0.79 (0.02)	0.73 (0.04)	**0.72 (0.13)**	**0.89 (0.05)**	**0.85 (0.06)**
DH270.1	0.66 (0.11)	0.92 (0.06)	0.83 (0.06)	**0.86 (0.05)**	**0.96 (0.02)**	**0.93 (0.03)**
DH270.5	0.70 (0.12)	0.92 (0.04)	0.84 (0.07)	**0.95 (0.03)**	**0.99 (0.01)**	**0.97 (0.02)**
DH270.6	0.74 (0.12)	0.93 (0.05)	0.87 (0.06)	**0.88 (0.09)**	**0.96 (0.02)**	**0.94 (0.05)**
PG16	0.23 (0.17)	0.80 (0.09)	0.85 (0.02)	**0.65 (0.13)**	**0.86 (0.07)**	**0.86 (0.05)**
PG9	0.17 (0.12)	0.80 (0.07)	**0.89 (0.01)**	**0.70 (0.09)**	**0.90 (0.04)**	0.88 (0.03)
PGDM1400	0.73 (0.04)	0.91 (0.04)	0.89 (0.02)	**0.82 (0.05)**	**0.93 (0.03)**	**0.92 (0.02)**
PGT121	0.57 (0.09)	0.87 (0.03)	0.82 (0.03)	**0.78 (0.04)**	**0.94 (0.02)**	**0.89 (0.02)**
PGT128	0.31 (0.08)	0.75 (0.11)	0.82 (0.01)	**0.73 (0.07)**	**0.90 (0.03)**	**0.87 (0.03)**
PGT135	0.37 (0.15)	0.78 (0.08)	0.72 (0.06)	**0.60 (0.04)**	**0.83 (0.03)**	**0.80 (0.03)**
PGT145	0.61 (0.07)	0.79 (0.05)	0.86 (0.02)	**0.75 (0.03)**	**0.90 (0.03)**	**0.89 (0.02)**
VRC26.08	0.53 (0.04)	0.88 (0.01)	0.82 (0.01)	**0.91 (0.05)**	**0.98 (0.02)**	**0.95 (0.03)**
VRC26.25	0.57 (0.09)	0.86 (0.04)	0.88 (0.02)	**0.82 (0.08)**	**0.95 (0.02)**	**0.92 (0.03)**
VRC38.01	0.46 (0.11)	**0.89 (0.05)**	0.80 (0.04)	**0.50 (0.11)**	0.80 (0.07)	**0.80 (0.04)**
Average	0.54 (0.09)	0.86 (0.05)	0.83 (0.03)	**0.77 (0.07)**	**0.92(0.03)**	**0.90 (0.03)**

gp41 MPER, gp41-gp120 interface, and fusion peptide						

2F5	0.72 (0.03)	0.93 (0.01)	0.86 (0.01)	**0.79 (0.04)**	**0.93 (0.02)**	**0.89 (0.02)**
35O22	0.33 (0.06)	**0.71 (0.03)**	0.68 (0.03)	**0.42 (0.06)**	0.71 (0.05)	**0.68 (0.02)**
4E10	0.00 (0.00)	0.68 (0.16)	**0.96 (0.00)**	**0.55 (0.08)**	**0.75 (0.05)**	0.92 (0.03)
8ANC195	**0.65 (0.08)**	**0.90 (0.05)**	**0.84 (0.04)**	0.50 (0.07)	0.77 (0.03)	0.73 (0.07)
PGT151	0.50 (0.13)	0.82 (0.07)	0.80 (0.04)	**0.68 (0.12)**	**0.87 (0.07)**	**0.85 (0.05)**
VRC34.01	**0.52 (0.10)**	**0.80 (0.05)**	**0.75 (0.05)**	0.50 (0.03)	0.74 (0.06)	0.73 (0.02)
Average	0.45 (0.07)	**0.81 (0.06)**	**0.81 (0.03)**	**0.57 (0.07)**	0.79 (0.04)	0.80 (0.03)

Global Average	0.43 (0.10)	0.83 (0.06)	0.83 (0.03)	**0.71 (0.07)**	**0.88 (0.04)**	**0.87 (0.03)**

*Note*: The numbers between the parentheses are the standard deviations (calculated with N-1 degrees of freedom) of the metrics recorded during the nested cross-validation. The metrics for SLAPNAP were obtained by running the script available from SLAPNAP (Williamson *et al.*, 2021a). The boldface values highlight the best metrics between SLAPNAP and our model.

## 5 Discussion

Our approach yields substantially better results in terms of averaged cross-validated metrics compared to the other methods: 0.75 versus 0.66 (bNAb-ReP) and 0.71 versus 0.43 (SLAPNAP) for MCC, 0.89 versus 0.84 (bNAb-ReP) and 0.88 versus 0.83 (SLAPNAP) for AUC, 0.89 versus 0.85 (bNAb-ReP) and 0.87 versus 0.83 (SLAPNAP) for accuracy. We recommend comparing the models by the MCC since it is a more discriminative metric that typically yields lower values. The nested cross-validation is a very stringent evaluation methodology and a computationally taxing one. The results agree with this, and we obtain slightly lower performance on this evaluation procedure, 0.71 versus 0.75 for MCC, 0.88 versus 0.89 for AUC, 0.87 versus 0.89 for accuracy. If we only pretrain (without fine-tuning for a specific antibody), we still achieve decent results, 0.55 to 0.57 MCC, 0.82 to 0.84 AUC, and 0.79 to 0.80 accuracy, as shown in Table 1. The variate models provide very similar results.

Despite the improvements in predictive performance, several aspects can be further explored and improved. The greatest drawback of our current model is that it is not explainable, unlike bNAb-ReP and SLAPNAP. Knowing the epitope/paratope is valuable and can provide insights into the mechanics of the antibody-antigen interaction. Furthermore, explainable models tend to be more trusted, and the analysis of the features might serve as an added validation. NNs usually require more effort to be made explainable, but this is something achievable. A possible solution to this challenge is Grad-CAM++ (Chattopadhay *et al.*, 2018). This method makes NNs explainable by considering the gradients that flow through the networks’ layers. An alternative is to use methods for finding feature importance that treat the model as a black-box, such as Williamson *et al.* (2021b) and Williamson *et al.* (2022). The Metropolis-Hastings algorithm from Yu *et al.* (2018) and Yu *et al.* (2019) is another method for feature selection which the authors combined with an SVR (Cortes and Vapnik, 1995; Drucker *et al.*, 1997). The SVR is used to evaluate the states sampled by the Metropolis-Hastings algorithm. However, we believe this method can be combined with other models as well, such as a NN. Another drawback of our model is that, at the moment, it does not tackle regression. However, this extension is also feasible. One challenge related to regression is the handling of the censored values, which are the values expressed as open intervals, such as ‘>50’. In the current work, we focused on recurrent networks and transformers; however, convolutional networks are another architecture that might be useful. Experimenting with convolutional NNs for modeling the antibody-virus interaction is a theme that could be explored in future works.

## 6 Code and data availability

The code and data underlying this article are available on Git and Zenodo (Dănăilă, 2022). The Git repository contains more branches:

1. main: shows an early version of our solution and is used to compute a baseline using the model from ICERI (Dănăilă and Buiu, 2021);
2. fc-att-fix: displays our model of choice FC-ATT-GRU, which combines fully-connected layers, attention and recurrent networks and was compared with bNAb-ReP (Rawi *et al.*, 2019);
3. fc-att-fix-nested-cv: compares the FC-ATT-GRU model with SLAPNAP (Williamson *et al.*, 2021a) using nested cross-validation;
4. 6cdr: shows the model and data processing based on CDRs;
5. 6cdr-trans: corresponds to the transformers architecture (Vaswani *et al.*, 2017); and
6. fc-att-ab-cls: contains the multitask-learning experiments.

The Zenodo project contains six versions that are snapshots of the branches from the Git repository. For enabling reproducible experiments, we created dataset partitions and saved them in our repository in JSON format. Other researchers can use those partitions in their experiments if they need to compare their models against ours using identical data. Those are found in the files splits_Rawi_comparison.json for the bNAb-ReP comparison, splits_SLAPNAP_ comparison_nested_cross_validation_X.json, where X is a number from one to five (each corresponding to one of the five folds of cross-validation), for the SLAPNAP comparison, and splits_uniform.json for hyperparameter tuning for pretraining. The partitions work together with a file named catnap_flat.json. This file represents the processed CATNAP dataset and contains tuples of four elements containing in order: record id, antibody id, virus id, binary ground truth. The dataset partitions refer to the ids of the records from the catnap_flat.json, more specifically to the first element from each tuple. We also store a snapshot of the CATNAP data at the time of this writing in the folder catnap_data on the branch fc-att-fix. Also, the CDR sequences and sequence offsets were saved in the file CDRs.json on the branch 6cdr.

The hyperparameters for different pretrained models are stored in the python package deep_hiv_ab_pred.hyperparameter in JSON format. We saved the hyperparameters for:

1. ICERI with all input combinations;
2. FC-ATT-GRU with one-hot encoded input;
3. FC-ATT-GRU with aminoacid properties input;
4. FC-ATT-GRU with both one-hot and aminoacid properties;
5. 6CDR-FC-GRU with aminoacid properties;
6. TRANSF with aminoacid properties; and
7. MULTITASK with aminoacid properties.

## 7 Conclusion

The main ideas of our article are to leverage both antibody and virus sequences to capture more generic relationships instead of focusing on specific antibodies, which may have less data available, and to take advantage of the full CATNAP dataset through NNs and transfer learning. It is known that NNs are versatile, and often, they may outperform the other types of algorithms on different tasks, especially when the available data is large. Nevertheless, their training and hyperparameter tuning are computationally expensive and complex. In the current work, we used a modern hyperparameter tuning method, the TPE (Bergstra *et al.*, 2011), to automate the process and find a suitable setup. We combined recurrent, fully-connected and attention layers to model the relationships between the antibody and virus sequences. We also looked into transformers and multitask-learning, but those did not bring any meaningful advantage. While the transformers architecture (Vaswani *et al.*, 2017) is considered state-of-the-art in natural language processing, for our given task, the data might be insufficient to derive benefits from this type of network. The aminoacids were expressed in multiple ways: static properties, one-hot encodings, or learned features. Overall, the static properties gave the best results and were also the most computationally efficient since they had a smaller dimension compared to the other approaches. Considering only the CDRs instead of the whole variable region for the antibody complicates the data preprocessing and provides a non-significant increase in predictive performance. Further research ideas are related to making the model explainable, investigating convolutional architectures, handling regression and censored data, finding additional data sources for network pretraining, and building a hybrid method that takes advantage of both sequence and structure data.

*Financial Support*: none declared.

*Conflict of Interest*: none declared.
